# Real-world implementation of pertuzumab from 2013 to 2023 for *de Novo* HER2-positive metastatic breast cancer in the Netherlands: a population-based study

**DOI:** 10.1016/j.breast.2026.104888

**Published:** 2026-07-25

**Authors:** Nan Ding, Sandra M.E. Geurts, Ingeborg J.H. Vriens, Renée Visserman, Thiemo J.A. van Nijnatten, Marjolein Smidt, Adri C. Voogd, Janneke Verloop, Vivianne C.G. Tjan-Heijnen

**Affiliations:** a Department of Medical Oncology, GROW Research Institute for Oncology and Reproduction, Maastricht University Medical Centre, Maastricht, the Netherlands; b Department of Radiology and Nuclear Medicine, GROW Research Institute for Oncology and Reproduction, Maastricht University Medical Centre, Maastricht, the Netherlands; c Dutch Expert Center for Screening (LRCB), Nijmegen, the Netherlands; d Department of Surgery, GROW Research Institute for Oncology and Reproduction, Maastricht University Medical Centre, Maastricht, the Netherlands; e Department of Epidemiology, Maastricht University, Maastricht, the Netherlands; f Department of Research and Development, Netherlands Comprehensive Cancer Organisation, Utrecht, the Netherlands

**Keywords:** Breast neoplasms, Neoplasm metastasis, Receptor, ErbB-2, Pertuzumab, Drug utilization, Socioeconomic factors

## Abstract

**Background:**

We aimed to describe the implementation of pertuzumab and identify factors associated with its use among patients with *de Novo* HER2-positive metastatic breast cancer (HER2+ MBC) in the Netherlands.

**Methods:**

Using Netherlands Cancer Registry data, we identified patients diagnosed with *de Novo* HER2+ MBC in 2013-2023 who received systemic therapy. Pertuzumab uptake by year of diagnosis was assessed, and segmented regression was used to estimate the time point at which a plateau was reached. Factors associated with pertuzumab use during the plateau period were evaluated using multivariable logistic regression, with adjusted odds ratios converted to adjusted relative risks (aRR).

**Results:**

Among 1,982 patients with *de Novo* HER2+ MBC, pertuzumab use increased from 9% in 2013 to 49% in 2014, 61% in 2016 and 78% in 2018, after which it plateaued at a rate of 78%. In 2018-2023, pertuzumab use was independently associated with age [50-74 years (aRR 0.75, 95% CI: 0.61-0.87) and ≥75 years (aRR 0.04, 95% CI: 0.02-0.07) vs < 50 years]; socioeconomic status [middle (aRR 0.88, 95% CI: 0.75-0.99) and low (aRR 0.80, 95% CI: 0.65-0.94) vs high]; hormone receptor status [positive (aRR 0.46, 95% CI: 0.34-0.60) vs negative disease]; and grade [3 (aRR 1.06, 95% CI: 1.01-1.10) vs 1-2].

**Conclusion:**

Pertuzumab uptake reached a plateau five years after approval of reimbursement in the Netherlands. Nowadays, around 80% of patients with *de Novo* HER2+ MBC received first-line pertuzumab-based therapy. Factors associated with pertuzumab non-use provide important insights for health policymakers aiming to ensure optimal access to effective cancer therapies.

## Highlights

• Pertuzumab uptake in *de Novo* HER2-positive metastatic breast cancer reached a plateau after five years in the Netherlands.
• During the plateau phase, around 80% of patients received first-line pertuzumab-based therapy.
• Pertuzumab uptake was lowest among patients aged ≥75 years (32%).
• Pertuzumab use was also lower in hormone receptor-positive tumours and patients with a middle or low socioeconomic status.

## Graphical abstract

**Figure:**
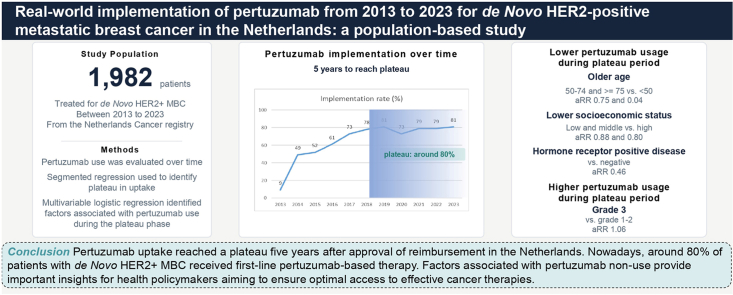

## 1 Introduction

*De Novo* HER2-positive metastatic breast cancer (HER2+ MBC), defined as metastatic disease at initial diagnosis, accounts for approximately 6-10% of all newly diagnosed HER2+ breast cancers and represents a clinically distinct subgroup within HER2+ MBC [1,2]. Although characterised by an aggressive tumour biology, *de Novo* HER2+ MBC has shown substantial improvements in survival over the past decade due to the development of increasingly effective HER2-targeted therapies [[3], [4], [5], [6]]. In the CLEOPATRA trial, the addition of pertuzumab to trastuzumab and docetaxel significantly prolonged overall survival, establishing this combination as the first-line standard of care [7]. Real-world studies have subsequently demonstrated survival outcomes approaching those reported in clinical trials, supporting the effectiveness of HER2-targeted therapy outside trial settings [8,9]. Current international guidelines continue to recommend pertuzumab-based therapy as the preferred first-line therapy for patients with HER2+ MBC [10].

More recently, additional HER2-targeted agents and combinations have expanded therapeutic options across earlier and later therapy lines. The PATINA trial demonstrated improved progression-free survival with the addition of endocrine therapy combined with palbociclib to maintenance trastuzumab and pertuzumab in HR+/HER2+ MBC [11]. Antibody-drug conjugates have further transformed the treatment landscape: trastuzumab-deruxtecan has shown marked efficacy compared with standard therapy in the DESTINY-Breast03 and DESTINY-Breast09 trials [12,13]. Further, new tyrosine kinase inhibitors such as tucatinib have shown benefit in the HER2CLIMB and HER2CLIMB05 trials [14,15]. These advances underscore the importance of monitoring and understanding real-world uptake of systemic therapies, not only for quality-of-care assessment but also for future budget impact analyses.

Pertuzumab received regulatory approval shortly after the publication of pivotal trial results, with approval by the U.S. Food and Drug Administration in July 2012, followed by the European Medicines Agency in March 2013 and national reimbursement approval in the Netherlands in July 2013. Despite this rapid approval process, translation of evidence into practice is rarely immediate or uniform. Real-world evidence (RWE) demonstrates variability in implementation due to institutional protocols, physician preferences, and patient factors [16]. While RWE often reports more modest population-level benefits than randomised controlled trials (RCTs), equitable delivery of effective therapies can yield substantial gains in selected subgroups [17]. A 100% uptake of a new drug is unrealistic, as clinical contraindications, comorbidities, or patient-related factors may preclude its use [[18], [19], [20]]. Real-world evidence studies can describe and explain patterns of use and non-use in routine clinical practice.

In this nationwide population-based study, we aimed to evaluate the real-world implementation of pertuzumab in the Netherlands after reimbursement approval. Specifically, we assessed temporal trends in pertuzumab use, determined the timing required to reach a stable implementation plateau, and identified patient- and tumour-related factors associated with its use. We focused on patients with *de Novo* HER2+ MBC, as treatment data in this population are routinely and comprehensively collected in a national database during the first year after breast cancer diagnosis, providing a unique opportunity to study real-world implementation patterns.

## 2 Patients and methods

### 2.1 Study design and data source

We conducted a nationwide, population-based, retrospective cohort study using data from the Netherlands Cancer Registry (NCR). The NCR records all newly diagnosed malignancies in the Netherlands based on notifications from the Nationwide Histopathology and Cytopathology Data Network and Pathology Archive (PALGA) and the National Registry of Hospital Discharges. Specially trained NCR data managers extract patient demographics, tumour characteristics, and treatment details during the first year after diagnosis, including systemic therapy regimens and start dates, from medical records.

### 2.2 Study population

We identified all patients diagnosed between 2013 and 2023 with *de Novo* HER2+ MBC, defined as the presence of distant metastases within three months of primary tumour diagnosis. HER2-positivity was defined as immunohistochemistry (IHC) 3+ or in situ hybridisation (ISH) amplification according to international guidelines. Patients were excluded if HER2 status was unknown, systemic treatment data were incomplete, or no systemic therapy was administered.

### 2.3 Variables

#### 2.3.1 Outcome

The primary outcome was receipt of pertuzumab as part of first-line systemic therapy, regardless of other agents. Treatment switches within 90 days were considered part of a sequential first-line regimen.

#### 2.3.2 Covariates

Patient characteristics included age (<50 years, 50-74 years, and ≥75 years), sex (male and female), socioeconomic status (SES: high, middle, low, and unknown), and region (A, B, C, D, E, F, G, and unknown). SES was based on median disposable household income per postal code area (CBS, 2019), adjusted for household size. Twelve original income categories were grouped into three (low, middle, high) to reduce overlap. SES reflects area-level, not individual, income and may vary within postcode areas. Cancer care in the Netherlands is organised into seven regional oncology networks, which were relabelled A through G to ensure anonymity [21].

Tumour characteristics included clinical T-stage (cT0, cT1-2, cT3-4, and cTx), clinical N-stage (cN0, cN1-3, and cNx), histology (non-specified type, lobular, and other), grade of the primary tumour (1-2, 3, and unknown), hormone receptor status of the primary tumour (negative, positive, and unknown), number of organs involved (single and multiple), and initial site of metastatic disease (bone only, soft tissue without visceral or central nervous system (CNS), visceral without CNS, CNS, and unknown). Hormone receptor (HR)-positive disease was defined as nuclear staining of more than 10% for oestrogen and/or progesterone receptors in the primary tumour.

### 2.4 Statistical analysis

Baseline patient and tumour characteristics for the entire study population were summarised using descriptive statistics. Categorical variables were reported as frequencies and percentages, while continuous variables, i.e. age, were summarised using the median and interquartile range (IQR).

Pertuzumab uptake over time was assessed by year of diagnosis, and segmented regression was used to estimate the year when use plateaued.

For patients diagnosed during the plateau period, baseline characteristics were also described. We used multivariable logistic regression to identify factors associated with pertuzumab use, e.g. age at diagnosis, SES, region, grade, hormone receptor status, number of organs involved, and initial metastatic site. Given the high prevalence of pertuzumab use (>10%), adjusted odds ratios (ORs) were converted to adjusted relative risks (aRRs) using the Zhang and Yu method [22]. Subgroups showing statistically significant differences in aRRs in the multivariable model were further examined to illustrate absolute differences in pertuzumab uptake. For these subgroups, adjusted probabilities of receiving pertuzumab were estimated using marginal predictions from a reduced multivariable logistic regression model that included only the significant covariates. Marginal probabilities were obtained on the response scale using the ‘emmeans’ package in R and represent population-standardised estimates, averaging predicted probabilities over the observed distribution of the remaining covariates in the study population. Ninety-five percent confidence intervals (CIs) were derived using model-based standard errors. For each subgroup, a p-value was obtained from a Chi-square test, reflecting whether pertuzumab use differed across the levels of that characteristic after multivariable adjustment. Adjusted probabilities and their 95% CIs were visualised using bar charts for ease of clinical interpretation.

Missing data were imputed using multiple imputations by chained equations, as implemented in R [23]. Estimates from the 20 imputed datasets were pooled using Rubin's rules to correctly account for missing-data uncertainty [24].

All statistical analyses were performed using R version 4.1.3 (R Core Team, Vienna, Austria). Figures were created using R version 4.1.3 (R Core Team, Vienna, Austria). A two-sided p-value <0.05 was considered statistically significant, while p-values <0.10 were interpreted as indicative of a trend.

## 3 Results

Between 2013 and 2023, a total of 163,076 patients were diagnosed with invasive breast cancer in the Netherlands, among whom 9644 (6%) presented with *de Novo* MBC (Supplementary Fig. 1). Of these, 2182 (23%) had *de Novo* HER2-positive MBC. After excluding 200 patients who had received best supportive care only, 1982 patients (90%) who received systemic therapy were included in this present study.

### 3.1 Baseline characteristics

The median age at diagnosis was 57 years (interquartile range: 47-68), with 32% aged under 50 years, 54% aged 50-74, and 14% aged 75 years or older (Table 1). Most patients were female (99%). SES was high in 33%, middle in 37%, and low in 30% of patients. The majority of tumours were non-specified type (91%), had grade 3 disease (51%) and were hormone receptor-positive (60%). At diagnosis, 54% of patients had a single organ involved. Metastatic sites included visceral metastases without CNS involvement (57%), bone-only metastases (25%), soft tissue metastases without visceral or CNS involvement (15%), and CNS metastases (3%). Key baseline characteristics before and after imputation are summarised in Supplementary Table 1.

**Table 1:** Patients and tumour characteristics in patients diagnosed and systemically treated for *de Novo* HER2+ metastatic breast cancer in the Netherlands in 2013-2023, n (%).

Characteristics	Total N = 1982
**Age at diagnosis**	
<50 years	638 (32)
50-74 years	1060 (54)
≥75 years	284 (14)
Median (IQR), years	57 (47-68)
**Sex**	
Female	1967 (99)
Male	15 (1)
**Socioeconomic status**	
High	651 (33)
Middle	724 (37)
Low	588 (30)
Unknown	N = 19
**Region**	
A	317 (16)
B	233 (12)
C	322 (16)
D	335 (17)
E	175 (9)
F	205 (10)
G	372 (19)
Unknown	N = 23
**cT-stage**	
cT0	27 (1)
cT1-2	934 (49)
cT3-4	956 (50)
cTx	N = 65
**cN-stage**	
cN0	227 (12)
cN1-3	1721 (88)
cNx	N = 34
**Histology**	
Non-specified type	1804 (91)
Lobular	143 (7)
Other	35 (2)
**Grade**^a^	
1-2	687 (49)
3	703 (51)
Unknown	N = 592
**Hormone receptor**^a^	
Negative	792 (40)
Positive	1184 (60)
Unknown	N = 6
**Number of organs involved**	
Single organ	1080 (54)
Multiple organs	902 (46)
**Initial metastatic site**	
Bone only	485 (25)
Soft tissue^b^	300 (15)
Visceral^c^	1131 (57)
CNS^d^	66 (3)

CNS = central nervous system, IQR = interquartile range, HER2+ = human epidermal growth factor receptor 2 positive, N = patients number, cN-stage = clinical nodal stage, cT-stage = clinical tumour stage. Percentages may not add up to or exceed 100% because of rounding. Unknown data were not included in the percentages.

a Of the primary tumour.

b Lymph nodes and skin, without visceral or CNS.

c Liver, lung, pancreas, heart, pleura, peritoneum, gastrointestinal track, kidney, endocrine glands, uterus and ovaries, without CNS.

d Brain, meninges, and eye.

### 3.2 Pertuzumab implementation over time

**Fig. 1:**
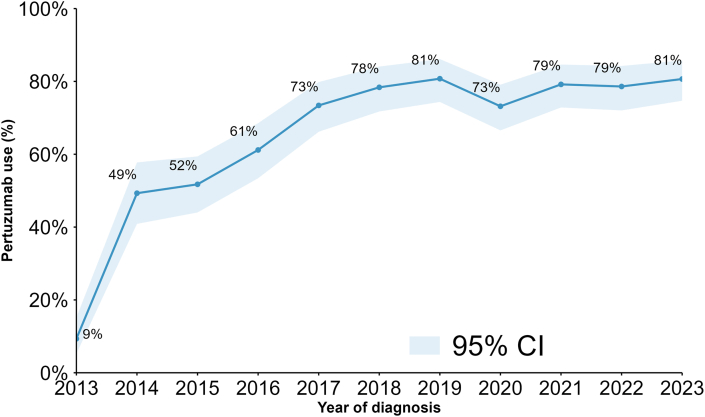
Pertuzumab implementation in patients systemically treated for *de Novo* HER2+ metastatic breast cancer in the Netherlands in 2013-2023.

Following its introduction in 2013, the percentage of patients receiving pertuzumab-based therapy increased from 9% in 2013 to 49% in 2014, 61% in 2016, and reached 78% in 2018, after which it fluctuated between 73% and 81% (Fig. 1). Among patients aged over 75 years, pertuzumab use increased from 0% in 2013 to 32% in 2019, followed by a transient decline to 7% in 2020 (likely due to the COVID pandemic) and a subsequent rise to 44% in 2021 (Supplementary Fig. 2). Such variability was not observed in younger patients and implementation patterns across other patient subgroups are also shown in Supplementary Fig. 2.

**Fig. 2:**
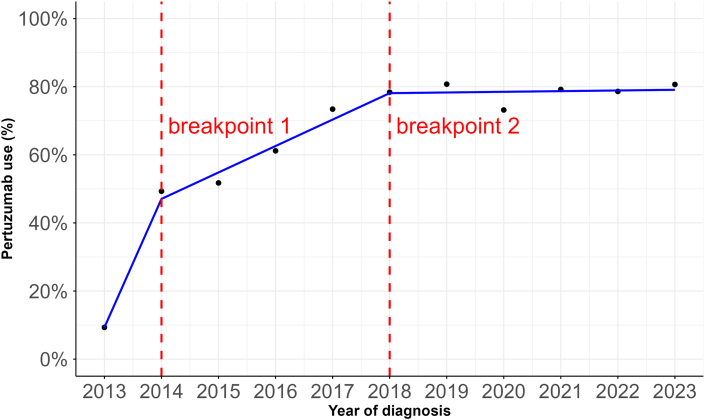
Segmented regression analysis showing the plateau in pertuzumab use rate in patients systemically treated for *de Novo* HER2+ metastatic breast cancer in the Netherlands in 2013-2023.

Segmented regression analysis showed a significant increase in pertuzumab use from 2013 to 2018, followed by a plateau from 2018 onward (Fig. 2). Baseline characteristics of 1173 patients diagnosed during the plateau period (2018-2023) are presented in Supplementary Table 2 and were largely comparable to those of the overall study population.

### 3.3 Pertuzumab use during the plateau period

**Fig. 3:**
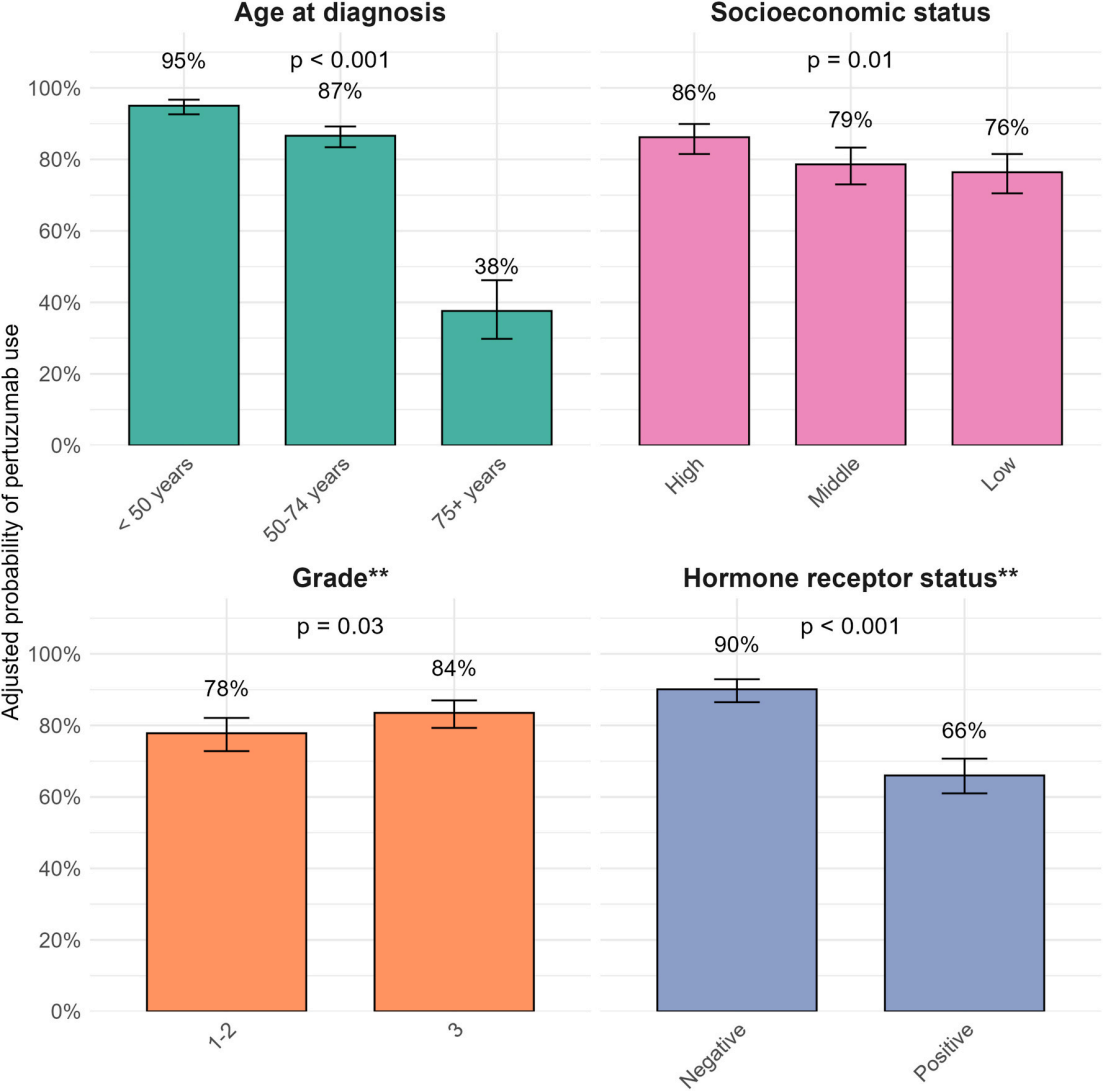
Adjusted probability of pertuzumab use by patient subgroups* in patients systemically treated for *de Novo* HER2+ metastatic breast cancer in the plateaued implementation phase (years 2018-2023) after its initial introduction in 2013 in the Netherlands *Only showing the subgroups with significant difference in pertuzumab use in Table 2. **Of the primary tumour.

In 2018-2023, pertuzumab was used in 93% of patients <50 years, 83% of those aged 50-74 years, and 32% of those aged over 75 years (Table 2). These differences were statistically significant after multivariable adjustment, with lower use in patients aged 50-74 years (adjusted relative risk (aRR) 0.75, 95% confidence interval (CI): 0.61-0.87) and especially in those over 75 years (aRR 0.04, 95% CI: 0.02-0.07) compared with patients <50 years (Table 2). This translates to an adjusted probability of pertuzumab use of 95% in patients aged <50 years, 87% of those aged 50-74 years and 38% of patients aged 75 years or over (Fig. 3). Pertuzumab use also varied by SES, decreasing from 84% in the high SES group to 79% in the middle and 72% in the low SES group (Table 2). After multivariable adjustment, both the middle and low SES groups remained significantly less likely to receive pertuzumab (aRR 0.88, 95% CI: 0.75-0.99 and aRR 0.80, 95% CI: 0.65-0.94, respectively) compared with the high SES group (Table 2). Corresponding adjusted probabilities were 86%, 79%, and 76%, respectively (p = 0.01) (Fig. 3). Similarly, tumour grade showed modest differences, with pertuzumab used in 75% of patients with grade 1-2 tumours and 82% of those with grade 3 tumours (Table 2). This difference persisted in the adjusted analyses, with slightly higher use among patients with grade 3 tumours (aRR 1.06, 95% CI: 1.01-1.10) (Table 2). Adjusted probabilities similarly demonstrated higher expected use in patients with grade 3 tumours than those with grade 1-2 tumours (84% vs 78%; p = 0.03) (Fig. 3). Pertuzumab was used in 91% of patients with hormone receptor negative disease and in 70% of those with hormone receptor positive disease (Table 2). After multivariable adjustment, hormone receptor positive disease was associated with lower pertuzumab use (aRR 0.46, 95% CI: 0.34-0.60) (Table 2). Adjusted probabilities for pertuzumab use were 90% for patients with hormone receptor-negative disease and 66% for those with hormone receptor-positive disease (p < 0.001) (Fig. 3). Among patients with HR + disease who did not receive pertuzumab, 34% received trastuzumab combined with endocrine therapy, 34% received endocrine therapy alone without HER2-targeted therapy, 29% received chemotherapy (with or without trastuzumab or endocrine therapy), and 3% received other therapy. Borderline difference was observed by region (p = 0.05, region F vs. region A) and no significant differences were observed by number of metastatic organs, or metastatic site (Table 2).

**Table 2:** Multivariable analysis of factors associated with pertuzumab use in patients systemically treated for *de Novo* HER2+ metastatic breast cancer in the Netherlands in 2018-2023.

	Number of patients	Pertuzumab use N (%)	Relative risk (95% CI), p-value	Adjusted relative risk^b^ (95% CI), p-value
**Age at diagnosis**				
<50 years	382	354 (93)	Ref.	Ref.
50-74 years	620	512 (83)	0.89 (0.85-0.93), <0.001	0.75 (0.61-0.87), <0.001
≥75 years	171	54 (32)	0.35 (0.28-0.44), <0.001	0.04 (0.02-0.07), <0.001
**Socioeconomic status**				
High	377	317 (84)	Ref.	Ref.
Middle	430	340 (79)	0.94 (0.88-0.99), 0.03	0.88 (0.75-0.99), 0.02
Low	366	263 (72)	0.91 (0.85-0.97), 0.01	0.80 (0.65-0.94), 0.004
**Region**				
A	186	146 (78)	Ref.	Ref.
B	131	108 (82)	1.00 (0.90-1.11), 0.97	1.00 (0.85-1.09), 0.97
C	195	158 (81)	0.95 (0.87-1.04), 0.25	0.95 (0.79-1.06), 0.41
D	202	154 (76)	0.97 (0.89-1.06), 0.52	0.96 (0.79-1.08), 0.55
E	113	92 (81)	1.00 (0.90-1.11), 0.95	0.99 (0.82-1.10), 0.82
F	123	85 (69)	0.90 (0.80-1.01), 0.07	0.78 (0.55-1.00), 0.05
G	223	177 (79)	0.97 (0.89-1.06), 0.49	0.97 (0.82-1.08), 0.67
**Grade**^a^				
1-2	594	443 (75)	Ref.	Ref.
3	579	477 (82)	1.06 (1.01-1.12), 0.02	1.06 (1.01-1.10), 0.03
**Hormone receptor status**^a^				
Negative	486	442 (91)	Ref.	Ref.
Positive	687	478 (70)	0.81 (0.77-0.85), <0.001	0.46 (0.34-0.60), <0.001
**Number of organs involved**				
Single organ	638	503 (79)	Ref.	Ref.
Multiple organs	535	417 (78)	0.95 (0.89-1.01), 0.11	0.9 (0.78-1.02), 0.10
I**nitial metastatic site**				
Bone only	290	215 (74)	Ref.	Ref.
Soft tissue^c^	171	140 (82)	1.06 (0.98-1.15), 0.14	1.07 (0.97-1.13), 0.16
Visceral^d^	671	536 (80)	1.05 (0.98-1.13), 0.19	1.06 (0.96-1.13), 0.20
CNS^e^	41	29 (70)	0.91 (0.74-1.12), 0.38	0.80 (0.48-1.09), 0.18

CI = confidence interval, CNS = central nervous system, HER2+ = human epidermal growth factor receptor 2 positive, N = number of patients, Ref. = reference.

a Of the primary tumour.

b Adjusted according to method of Zhang and Yu.

c Lymph nodes and skin, without visceral or CNS.

d Liver, lung, pancreas, heart, pleura, peritoneum, gastrointestinal track, kidney, endocrine glands, uterus and ovaries, without CNS.

e Brain, meninges, and eye.

## 4 Discussion

Once a new treatment has shown to be effective, national reimbursement and guideline update procedures are initiated. In this present study, we aimed to determine how long it would take to complete the implementation of pertuzumab use in a real-world setting after re-imbursement was approved. In this nationwide, population-based cohort of 1982 patients with *de Novo* HER2+ MBC in the Netherlands, pertuzumab use initially increased rapidly after its approval in 2013, but plateauing only five years later, by 2018. A temporal decline was observed in 2020, followed by recovery in subsequent years, consistent with a temporary disruption in oncology care delivery during the COVID-19 pandemic rather than a sustained change in clinical practice. At the plateau period approximately 80% of systemically treated patients received first-line pertuzumab. In the plateau period, we observed lower pertuzumab use among older patients, those with lower SES, and those with hormone receptor-positive disease. Although a small statistically significant difference was also observed according to tumour grade, this association was modest and is unlikely to reflect clinically meaningful variation in treatment decision-making. Overall, our findings indicate that pertuzumab use became established over time, while relevant differences in uptake across patient subgroups remained.

Earlier real-world studies have primarily examined the impact of pertuzumab-based therapy on survival outcomes in patients with MBC, rather than on its implementation [[25], [26], [27], [28]]. For example, analyses from the Dutch SONABRE Registry showed that pertuzumab was used in approximately 48% of patients treated in 2013-2017 and that median overall survival improved from 28.3 months in 2008-2012 to 39.7 months after the introduction of pertuzumab [25]. Using the same registry, a subsequent analysis stratified by treatment line found that median OS declined progressively across the first four lines of therapy. Notably, median OS was not reached at 56.0 months among patients treated with first-line pertuzumab, trastuzumab, and chemotherapy, compared with markedly shorter survival in later treatment lines [26]. Similar real-world improvements in survival have been reported in studies from the NCR, the French ESME cohort, and Canadian population-based datasets, all demonstrating substantial gains following the uptake of pertuzumab-based first-line therapy [[27], [28], [29]]. However, none of these studies explicitly evaluated the implementation process itself, especially when pertuzumab use reached a stable plateau in routine practice. Understanding this aspect is essential for interpreting real-world outcomes and for informing future health-care planning and budget impact assessments.

Obviously, a pertuzumab uptake of less than 100% in the steady-state situation does not indicate suboptimal care. International guidelines emphasize that treatment choices in MBC should be individualized according to patient factors, such as frailty by comorbidities, and patient preferences [10,[30], [31], [32], [33]]. These principles support that not all patients are candidates for pertuzumab-based systemic therapy, even in settings with universal access.

In our study, age was shown to be the strongest determinant of pertuzumab use, with substantially lower uptake observed among older patients compared with younger age groups. This age-related difference in treatment use is notable given that, in the CLEOPATRA phase III trial, the subgroup of patients aged ≥65 years experienced a robust progression-free survival benefit from pertuzumab addition (HR = 0.52; 95% CI: 0.31-0.86), comparable to those aged <65 (HR = 0.65; 95% CI: 0.53-0.80), yet only 16% of participants were 65+ years of age. In general, older patients remain under-represented in pivotal trials [33]. Dedicated studies, such as the EORTC 75111-10114 trial in patients ≥70 or frail ≥60, confirmed feasibility of pertuzumab-based therapy, but also highlighted toxicity concerns and frequent dose adjustments [34]. Population-based cohorts from Canada and Europe demonstrated reduced prescription of HER2-targeted therapies with advancing age [8,9]. These patterns likely reflect physician concerns about frailty, comorbidities, and treatment tolerability in older patients, which may lead to more conservative treatment selection in routine clinical practice. Given the aging population and rising incidence of MBC in older adults, this treatment gap may have important implications for population-level outcomes.

We also identified socioeconomic disparities, with patients from lower-income areas significantly less likely to receive pertuzumab. This mirrors findings from England's NHS, and from the United States [35,36]. Several mechanisms may contribute to these socioeconomic differences in treatment uptake. Lifestyle-related risk factors such as smoking, obesity, and physical inactivity are more common in lower-SES populations and are associated with a higher comorbidity burden and reduced treatment tolerance, which may limit eligibility for pertuzumab in routine clinical practice [37,38]. In addition, patients with lower SES may be less proactive in navigating the healthcare system, potentially limiting their engagement in shared decision-making about HER2-targeted therapy. Using plain language and allowing more time for shared decision-making with this patient group may help. Additional support from nursing staff during this process may also help patients make more informed treatment decisions. [39]. In addition, indirect financial barriers, including travel distance, transportation needs, and parking costs, may further impede access to hospital-based systemic therapies, particularly for patients with limited financial resources [40]. Together, these factors suggest that differences in lifestyle-related health status and comorbidity burden play a central role in shaping real-world pertuzumab use, while system-level factors such as referral pathways and institutional practice variation may further contribute. Notably, socioeconomic disparities in cancer care are not specific to HER2-targeted therapy or breast cancer, but reflect a broader and complex pattern of health inequality for which no single intervention is likely to be sufficient. Hormone receptor status further influenced treatment decisions, with lower pertuzumab use in hormone receptor-positive disease. This pattern may reflect clinician preference for first-line endocrine-based regimens without HER2 targeted therapy, despite guidelines endorsing dual HER2 blockade across hormone receptor subgroups [41]. Indeed, a substantial proportion of patients with HR + disease who did not receive pertuzumab were treated with endocrine-based regimens, either combined with trastuzumab or alone. In the CLEOPATRA trial, pertuzumab improved overall survival in both subgroups, with comparable benefit in patients with hormone receptor negative disease (HR = 0.64; 95% CI: 0.50-0.82) as with hormone receptor positive disease (HR = 0.74; 95% CI: 0.58-0.96) [4]. PERTAIN and SYSUCC-002 demonstrated that endocrine therapy combined with HER2-targeted therapy can be an effective alternative to chemotherapy-based first-line therapy in selected patients [42,43]. More recently, the PATINA trial demonstrated that adding endocrine therapy and palbociclib to maintenance pertuzumab and trastuzumab further improved progression-free survival in HR+/HER2+ MBC, underscoring the continued importance of pertuzumab-based dual HER2 blockade even when endocrine therapy is incorporated into the treatment strategy [11]. Regardless the backbone discussion, omitting dual HER2 blockade bears the risk of compromising long-term outcome of patients with HER2+ MBC.

This study contributes important real-world evidence that complements findings from clinical trials by illustrating how HER2-targeted therapies like pertuzumab are implemented across a broad and diverse patient population. Unlike trial settings, real-world data capture the complexities of clinical practice, including variations driven by patient-, tumour-, and healthcare system factors [44,45]. Our population-based analysis sheds light on how such variables influence treatment decisions. Notably, our findings show that substantial gaps in implementation can persist for years even after an effective therapy has received reimbursement approval, underscoring that underutilization does not resolve automatically over time. These insights are particularly valuable for health policymakers aiming to ensure optimal access to and equitable implementation of costly cancer therapies.

This study offers several strengths. We utilized a comprehensive, population-level dataset encompassing all patients treated for *de Novo* HER2+ MBC across a decade, enhancing the generalizability of our findings. Additionally, the longitudinal nature of the data enabled evaluation of temporal adoption patterns, adding a dynamic component to our understanding of implementation. However, certain limitations warrant consideration. The retrospective design introduces potential for residual confounding, as important clinical parameters such as WHO performance status, comorbidity, and patient preferences were not captured. Some variables, including tumour grade and socioeconomic status, had incomplete data, for which we used imputation which may not be exactly the same as in reality would have been. Moreover, information on HER2 testing methodology was not available. These gaps limit our ability to assess real-world implementation and may have introduced misclassification bias, particularly in earlier years of the study. Additionally, as recurrent MBC is not captured with the same completeness in the NCR as *de Novo* disease, our study was restricted to the latter. Our related SONABRE registry work, which includes both *de Novo* and recurrent HER2+ advanced breast cancer, similarly showed modest pertuzumab implementation suggesting the slow implementation observed here is unlikely to be specific to *de Novo* disease [46]. Nevertheless, the high completeness and consistency of the NCR data for *de Novo* MBC ensure that the findings remain robust and informative.

## 5 Conclusion

In conclusion, by 2018, pertuzumab use in the Netherlands reached a plateau, five years after its approval, in four-fifths of systemically treated patients with *de Novo* HER2+ MBC. Non-use was mainly seen in older patients, those with lower SES, and hormone receptor-positive disease. Although a complete implementation is not feasible nor desirable, efforts should be made to fasten up the implementation process to ensure that advances in HER2-targeted therapies are delivered equitably to all eligible patients.

## Data sharing statement

Data are available from the Netherlands Comprehensive Cancer Organisation upon reasonable request and subject to approval.

## Ethics statement

The study was approved by the Privacy Committee of the NCR. The use of anonymised data does not require informed consent under Dutch Civil Law.

## Funding

This research did not receive any specific grant from funding agencies in the public, commercial, or not-for-profit sectors.

## CRediT authorship contribution statement

**Nan Ding:** Conceptualization, Data curation, Formal analysis, Investigation, Methodology, Validation, Visualization, Writing – original draft, Writing – review & editing. **Sandra M.E. Geurts:** Conceptualization, Investigation, Methodology, Validation, Writing – review & editing. **Ingeborg J.H. Vriens:** Writing – review & editing. **Renée Visserman:** Writing – review & editing. **Thiemo J.A. van Nijnatten:** Writing – review & editing. **Marjolein Smidt:** Writing – review & editing. **Adri C. Voogd:** Writing – review & editing. **Janneke Verloop:** Resources, Writing – review & editing. **Vivianne C.G. Tjan-Heijnen:** Conceptualization, Investigation, Methodology, Supervision, Validation, Writing – review & editing.

## Declaration of competing interest

The authors declare the following financial interests/personal relationships which may be considered as potential competing interests: Vivianne C.G. Tjan-Heijnen reports a relationship with Novartis that includes: funding grants. Vivianne C.G. Tjan-Heijnen reports a relationship with Gilead Sciences Inc that includes: funding grants. Vivianne C.G. Tjan-Heijnen reports a relationship with Eli Lilly that includes: funding grants. Vivianne C.G. Tjan-Heijnen reports a relationship with Roche that includes: funding grants. Vivianne C.G. Tjan-Heijnen reports a relationship with Pfizer that includes: funding grants. Vivianne C.G. Tjan-Heijnen reports a relationship with AstraZeneca that includes: funding grants. Vivianne C.G. Tjan-Heijnen reports a relationship with Daiichi Sankyo Inc that includes: funding grants. Thiemo J.*A. van* Nijnatten reports a relationship with Hologic Inc that includes: speaking and lecture fees. Thiemo J.*A. van* Nijnatten reports a relationship with Bayer Healthcare that includes: board membership, funding grants, and speaking and lecture fees. Thiemo J.A. van Nijnatten reports a relationship with GE Healthcare that includes: board membership, funding grants, and speaking and lecture fees. Thiemo J.A. van Nijnatten reports a relationship with Screenpoint Medical that includes: consulting or advisory. Ingeborg J.H Vriens reports a relationship with AstraZeneca that includes: funding grants. Ingeborg J.H Vriens reports a relationship with Pfizer that includes: funding grants. Ingeborg J.H Vriens reports a relationship with Novartis that includes: funding grants. Ingeborg J.H Vriens reports a relationship with Eli Lilly and Company that includes: funding grants. Sandra M.E. Geurts reports a relationship with AstraZeneca that includes: funding grants. Sandra M.E. Geurts reports a relationship with DAIICHI SANKYO COMPANY, LIMITED that includes: funding grants. Sandra M.E. Geurts reports a relationship with Eli Lilly that includes: funding grants. Sandra M.E. Geurts reports a relationship with Menarini Stemline UK Limited that includes: funding grants. Sandra M.E. Geurts reports a relationship with Merck Sharp & Dohme UK Ltd that includes: funding grants. Sandra M.E. Geurts reports a relationship with Novartis that includes: funding grants. Sandra M.E. Geurts reports a relationship with Pfizer that includes: funding grants. Sandra M.E. Geurts reports a relationship with Seagan that includes: funding grants. Sandra M.E. Geurts reports a relationship with Gilead that includes: funding grants. Nan Ding reports a relationship with China Scholarship Council that includes: funding grants. If there are other authors, they declare that they have no known competing financial interests or personal relationships that could have appeared to influence the work reported in this paper.
