# Supplementary material for: Real-world implementation of pertuzumab from 2013 to 2023 for de Novo HER2-positive metastatic breast cancer in the Netherlands: a population-based study
Source: Breast. 2026 Jul 25;89:104888. doi: 10.1016/j.breast.2026.104888 (PMC13450629; doi:10.1016/j.breast.2026.104888)
Supplement: Supplementary file 1 [file mmc1.docx]

**Supplementary materials for “Real-world implementation of pertuzumab from 2013 to 2023 for *de Novo* HER2-positive metastatic breast cancer in the Netherlands: a population-based study”**

**Supplementary Figure 1.** Study flow chart

N= number of patients , HER2= human epidermal growth factor receptor 2

**Supplementary Table 1.** Key baseline characteristics before and after multiple imputation, in patients diagnosed and systemically treated for *de Novo* HER2+ metastatic breast cancer in the Netherlands in 2013-2023

|  | **Before imputation** | **After imputation** |
| --- | --- | --- |
| **Characteristics** | N=1982 | |
| **Region** |  |  |
| A | 317 (16) | 320 (16) |
| B | 233 (12) | 235 (12) |
| C | 322 (16) | 330 (17) |
| D | 335 (17) | 339 (17) |
| E | 175 (9) | 176 (9) |
| F | 205 (10) | 208 (10) |
| G | 372 (19) | 374 (19) |
| Unknown | N=23 | N=0 |
| **Socioeconomic status** |  |  |
| High | 651 (33) | 656 (33) |
| Middle | 724 (37) | 730 (37) |
| Low | 588 (30) | 596 (30) |
| Unknown | N=19 | N=0 |
| **Grade*** |  |  |
| 1-2 | 687 (49) | 976 (49) |
| 3 | 703 (51) | 1006 (51) |
| Unknown | N=592 | N=0 |
| **Hormone receptor status*** |  |  |
| Positive | 1184 (60) | 1190 (60) |
| Negative | 792 (40) | 792 (40) |
| Unknown | N=6 | N=0 |

N = number of patients. *Of the primary tumour. Percentages may not add up to or exceed 100% because of rounding.

**Supplementary Figure 2.** Pattern of implementation of pertuzumab use by A) age, B) social economic status, C) regions, D) grade, E) hormone receptor status, F) number of organs involved, G) metastatic site in patients diagnosed and systemically treated for *de Novo* HER2+ metastatic breast cancer in the Netherlands in 2013-2023

*Of the primary tumour.

**Supplementary Table 2.** Patients and tumour characteristics in patients diagnosed and systemically treated for *de Novo* HER2+ metastatic breast cancer in the Netherlands in 2018-2023

| **Characteristics** | **Total**  **N=1173** |
| --- | --- |
| **Age at diagnosis** |  |
| < 50 years | 382 (33) |
| 50-74 years | 620 (53) |
| ≥ 75 years | 171 (15) |
| Median (IQR) | 56 (47-69) |
| **Sex** |  |
| Female | 1163 (99) |
| Male | 10 (1) |
| **Socioeconomic status** |  |
| High | 373 (32) |
| Middle | 427 (37) |
| Low | 362 (31) |
| Unknown | N=11 |
| **Region** |  |
| A | 186 (16) |
| B | 130 (11) |
| C | 195 (17) |
| D | 202 (17) |
| E | 113 (10) |
| F | 123 (10) |
| G | 223 (19) |
| Unknown | N=1 |
| **Clinical T-stage** |  |
| cT0 | 14 (1) |
| cT1-2 | 548 (48) |
| cT3-4 | 572 (50) |
| cTx | N=39 |
| **Clinical N-stage** |  |
| cN0 | 127 (11) |
| cN1-3 | 1028 (89) |
| cNx | N=18 |
| **Histology** |  |
| Ductal | 1061 (91) |
| Lobular | 84 (7) |
| Other | 28 (2) |
| **Grade*** |  |
| 1-2 | 508 (51) |
| 3 | 479 (49) |
| Unknown | N=186 |
| **Hormone receptor*** |  |
| Negative | 486 (41) |
| Positive | 686 (59) |
| Unknown | N=1 |
| **Number of organs involved** |  |
| Single organ | 638 (54) |
| Multiple organs | 535 (46) |
| **Initial metastatic site** |  |
| Bone only | 290 (25) |
| Soft tissue^1^ | 171 (15) |
| Visceral^2^ | 671 (57) |
| CNS^3^ | 41 (4) |

CNS= central nervous system, HER2+= human epidermal growth factor receptor 2 positive, N= patients number, N-stage= nodal stage, T-stage= tumour stage. Percentages may not add up to or exceed 100% because of rounding. Unknown data were not included in the percentages. *Of the primary tumour, ^1^Lymph nodes and skin, without visceral or CNS, ^2^Liver, lung, pancreas, heart, pleura, peritoneum, gastrointestinal track, kidney, endocrine glands, uterus and ovaries, without CNS, ^3^Brain, meninges, and eye.
